# Willingness to Receive the COVID-19 Vaccination and the Psychological State of Japanese University Students: A Cross-Sectional Study

**DOI:** 10.3390/ijerph19031654

**Published:** 2022-01-31

**Authors:** Shogo Tsutsumi, Noriaki Maeda, Tsubasa Tashiro, Satoshi Arima, Rami Mizuta, Kazuki Fukui, Koichi Naito, Makoto Komiya, Yukio Urabe

**Affiliations:** 1 Graduate School of Biomedical and Health Sciences, Hiroshima University, Hiroshima 734-8553, Japan; shogo-tutumi@hiroshima-u.ac.jp (S.T.); norimmi@hiroshima-u.ac.jp (N.M.); tsubasatashiro716@hiroshima-u.ac.jp (T.T.); satoshi-arima4646@hiroshima-u.ac.jp (S.A.); m202263@hiroshima-u.ac.jp (R.M.); kazuki-fukui@hiroshima-u.ac.jp (K.F.); makoto-komiya@hiroshima-u.ac.jp (M.K.); 2 Department of Health Science, Nagoya Women’s University, Nagoya 467-8610, Japan; kouichi2007creha@yahoo.co.jp

**Keywords:** coronavirus disease, COVID-19, vaccine, university student, psychological distress, Japan

## Abstract

Vaccinations may be one of the solutions to end the COVID-19 pandemic. One’s psychological state may be strongly related to one’s willingness to be vaccinated. This study investigated the relationship between the psychological state of Japanese university students and their willingness to be vaccinated. A self-report questionnaire on COVID-19, its vaccines (vaccination status, and perceived efficacy and safety), and psychological state (anxiety and depressive mood) was administered online, and 560 valid responses were obtained. The unvaccinated group reported significantly lower perceived vaccine effectiveness and importance than the vaccinated group. However, there were no differences in anxiety and depressive mood symptoms between the two groups. Multivariate logistic regression analysis was performed on the unvaccinated participants to identify the factors associated with their unwillingness to be vaccinated; there was a significant association between anxiety and unwillingness to receive the vaccine (*p* < 0.05). However, there was no significant association between depressive mood and unwillingness to receive the vaccine. The results suggest that timely psychological support for Japanese university students experiencing high levels of anxiety is important in accelerating vaccination programs.

## 1. Introduction

COVID-19 is a severe respiratory disease caused by the SARS-CoV-2 virus. It was declared a pandemic by the World Health Organization (WHO) on 11 March 2020 [1]. In Japan, the government has declared a state of emergency four times so far, imposing several restrictions each time on its citizens. The COVID-19 pandemic has negatively impacted people’s mental health, leading to conditions such as anxiety and depression [2,3]. Thus, a vaccine for COVID-19 could not only be a promising solution to end the pandemic but could also help restore people’s mental health and daily lives.

Several countries have reported their citizens’ varying willingness to receive COVID-19 vaccinations [4]. Existing literature indicates that Japanese people have low perceived safety and efficacy of the vaccine as compared to other countries [4]. Japanese people have also shown a greater lack of trust in vaccines as compared to other countries [5]. There is a deep-rooted skepticism about the efficacy of vaccines as a countermeasure against infectious diseases in Japan [6,7]. Given that the conditions for vaccination may differ across countries, it is necessary to examine the causes of vaccine hesitancy; that is, a delay in or refusal of being vaccinated, despite the availability of vaccination services [7].

Vaccination is one of the most effective ways to build herd immunity against COVID-19. Therefore, it is necessary to expand vaccination programs across all age groups, without a generational bias [8]. Meanwhile, studies have shown that acceptance of the vaccine among the younger generations is lower than among the older generations [9,10]. In fact, the vaccination rate of older adults in Japan (40–80 years) is 77.0%, whereas that of the youth (under 40 years) is 44.3%. This indicates a considerable difference in the vaccination rates between generations despite the availability of vaccination services [11]. It also reflects the youth’s tendency to believe that they are immune to COVID-19 [12]. Thus, to achieve herd immunity, it is important to understand the factors influencing the acceptance of vaccination among young people.

Factors influencing COVID-19 vaccine acceptance and the intention to be vaccinated include individuals’ perceptions of the benefits and efficacy of the vaccine [13,14], and their psychological state [15]. One study suggested that vaccine hesitancy among university students is associated with psychological factors, and that an intervention targeting these factors may increase vaccination rates [15]. Moreover, although an understanding of the willingness to be vaccinated may assist in the dissemination of vaccination programs, the intention to be vaccinated may change over time [16,17]. For example, research investigating the willingness to follow up with the COVID-19 vaccination showed that 74.1% of the participants were willing to receive a vaccine in April 2020, whereas only 56.2% were willing to do so in December 2020 (seven months later) [16]. Although the reasons for this phenomenon are unclear, it is possibly because they had been exposed to various speculations and media reports amid rapid changes in the infectious situation. However, to the best of our knowledge, no reports exist on the relationship between the psychological state of young people in Japan and their willingness to be vaccinated after vaccination services became available.

Thus, this study aimed to determine the effect of the psychological state of university students in Japan on their willingness to receive the COVID-19 vaccine. The hypothesis was that the students with high levels of anxiety and depressive mood symptoms would have low willingness to be vaccinated.

## 2. Materials and Methods

### 2.1. Study Design and Participants

This cross-sectional study, with an observational design, was performed electronically from 30 July 2021, to 13 September 2021. It was conducted in compliance with the recommendations of the Strengthening the Reporting of Observational Studies in Epidemiology (STROBE) statement [18] and according to the Checklist for Reporting Results of Internet E-Surveys (CHERRIES) statement [19]. A previous study that surveyed Japanese people on their attitudes toward vaccination suggested that 45.5% of younger people aged between 20 and 49 years reported being unwilling to be vaccinated [20]. Based on this and a previous report by the Prime Minister’s Office of Japan [11], we set a target sample size of about 100 people who are unwilling to be vaccinated, aimed for a response rate of 40%, and projected that we would need to distribute the questionnaire to 1000 people. We contacted university faculty members who are affiliated with educational institutions in Japan and asked them to distribute the link to the questionnaire within their organizations. About four weeks after the distribution, we sent a reminder. The following information was given to the respondents before they began the survey: the questionnaire could be completed anonymously and each person should respond just once. Questionnaires were sent to 1000 university students in Japan via Google Forms (Alphabet, Mountain View, CA, USA), and 577 responses were received. The inclusion criteria were university students who consented to participate in the study. Among them, 17 students who took psychiatric drugs every day or provided insufficient responses were excluded from the analysis. Completed questionnaires from 560 students were retained for analysis. This study was conducted in accordance with the guidelines proposed in the Declaration of Helsinki and was approved by the Epidemiology Ethics Committee of Hiroshima University (approval ID: E-2531).

### 2.2. Questionnaire

We asked four questions on basic demographic information, nine questions about COVID-19-related information and COVID-19 vaccines, and four questions about psychological distress.

#### 2.2.1. Social Demographics

We recorded different participant characteristics, including age, gender, household structure (living alone or with others), and part-time job (employed or unemployed).

#### 2.2.2. COVID-19-Related Information and COVID-19 Vaccines

The questions about COVID-19 and its vaccines comprised behavioral restrictions due to COVID-19, fear and knowledge of COVID-19, experience of COVID-19 vaccination, willingness to be vaccinated, and attitudes toward vaccination.

Movement restrictions due to COVID-19 were classified as “strict confinement”, “confinement except for basic shopping or work”, and “no confinement”. Participants indicated their fear of COVID-19 by checking “Yes” or “No”. The two questions to determine knowledge of COVID-19 were as follows: (1) Is there a possibility of being reinfected after recovering from COVID-19 infection? and (2) Does an effective vaccine against COVID-19 currently exist [21]? The COVID-19 vaccination status was coded as “Yes” (including both one and two doses received) and “No” (zero doses received). Unvaccinated participants were further required to state their willingness to be vaccinated in the future with a “Yes” or “No” response. Attitudes toward vaccination were assessed by two questions pertaining to the perceived importance of vaccination in safeguarding oneself and others [21]. Responses were rated on a five-point Likert scale, ranging from 1 (‘not at all’) to 5 (‘very important’), such that high scores indicated greater perceived importance of the vaccine.

#### 2.2.3. Psychological State

The questionnaire included two initial screening tools—the Generalized Anxiety Disorder 2-item inventory (GAD-2) and the Patient Health Questionnaire (PHQ-2)—to measure the frequency of anxiety symptoms [22,23] and depressive mood [22,24] over the past two weeks, respectively. The responses to each item were rated on a four-point scale ranging from 0 (‘not at all’) to 3 (‘nearly every day’), with a maximum total score of 6 for both tools. For each scale, a score of ≥3 was considered a positive screening [23].

### 2.3. Statistical Analysis

In Model 1, the respondents were divided into two groups, namely the “Vaccinated group” and the “Unvaccinated group” based on their vaccination status. A secondary analysis was conducted in Model 2 wherein the respondents in the “Unvaccinated group” were further grouped as “Willing to receive the vaccine” and “Not willing to receive the vaccine” based on their willingness to be vaccinated.

In Model 1, the vaccinated and unvaccinated groups were analyzed for differences in psychological state, COVID-19-related information, and COVID-19 vaccines using Mann–Whitney U and Chi-Squared tests. Using the same tests in Model 2, the groups with and without the willingness to receive a vaccine were analyzed for differences in psychological state, COVID-19-related information, and COVID-19 vaccines.

In Model 2, multiple logistic regression analysis was conducted to identify the psychological factors associated with the willingness to receive the vaccine. Unvaccinated participants who were willing to receive the vaccine were coded as “1”, and those who were not willing to receive the vaccine were coded as “0”. A Wald test was conducted to eliminate variables with *p* > 0.05. The possibility of multicollinearity of the independent variables was evaluated using the variance inflation factor. All data were tested for significance at *p* < 0.05 and were analyzed using the IBM SPSS Statistics for Windows (version 23.0; IBM Corp., Armonk, NY, USA).

A previous study suggested that the number of participants per variable should be greater than or equal to 10 [25]. Because the sample size for the logistic regression analysis was established using four independent parameters, 10 times the number of participants of those parameters was required per group. Therefore, at least 40 participants were needed in each group (willing to receive the vaccine and not willing to receive the vaccine).

## 3. Results

**Figure 1:**
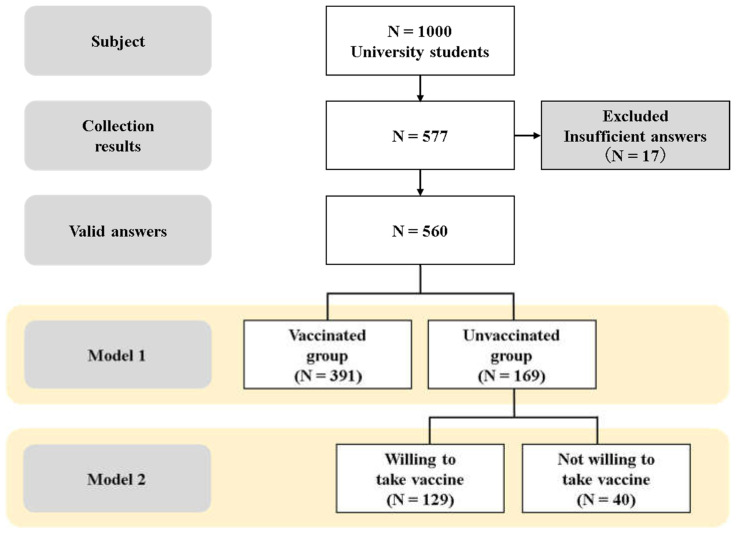
Flowchart of participant recruitment.

A total of 577 responses were obtained, of which 17 were excluded. Thus, 560 participants were included in the study (Figure 1). The response rate was 56%.

### 3.1. Comparison between the Vaccinated and Unvaccinated Groups

Table 1 presents the sociodemographic data of the participants according to their vaccination status. Of the 560 participants (mean (M_age_) = 21.4 years, standard deviation (SD) = 3.1 years), 61.1% were females, and 69.8% reported that they had already been vaccinated. Furthermore, approximately half of the participants in both groups lived alone. More than 70% of the participants in both groups had a part-time job.

The unvaccinated group exhibited lower perceived effectiveness of the COVID-19 vaccine than the vaccinated group (*p* = 0.015, effect size = 0.103). Regarding the perceived importance of vaccination to protect oneself, the median for the vaccinated group was 5.0, whereas that for the unvaccinated group was 4.0 (*p* < 0.01, effect size = −0.326). Regarding the importance of vaccination to protect others, the median for the vaccinated group was 5.0 and that for the unvaccinated group was 4.0 (*p* < 0.01, effect size = −0.224). However, there were no significant differences in confinement due to the pandemic or in fear of infection between the two groups. Moreover, there were no significant differences in the perceived risk of reinfection or in the mental health-related parameters of anxiety and depressive mood between the two groups.

### 3.2. Differences in the Willingness to Receive the Vaccine among the Unvaccinated Participants

Table 2 presents the categorization of participants in the unvaccinated group based on their willingness to receive the vaccine. In this section, the group with the participants who were willing to receive the vaccine is referred to as Group A, and the group with the participants who were unwilling to receive the vaccine is referred to as Group B.

There were no significant differences in age, gender, household structure, employment status, and movement restriction status between the two groups. The perceived effectiveness of an existing vaccine was significantly lower in Group B as compared to Group A (*p* = 0.001, effect size = 0.259). Regarding the perceived importance of vaccination to protect oneself, the medians for Group A and Group B were 4.0 and 3.0, respectively (*p* < 0.01, effect size = −0.528). For perceived importance of vaccination to protect others, the medians for Group A and Group B were 5.0 and 3.0, respectively (*p* < 0.01, effect size = −0.536). Regarding psychological distress, 17 (13.2%) in Group A and 12 (30.2%) in Group B screened positive for anxiety symptoms (*p* < 0.05), and 13 (10.1%) in Group A and 5 (12.5%) in Group B screened positive for depressive symptoms (*p* = 0.664).

### 3.3. Association of Unwillingness to Receive the Vaccine with Psychological State

In this study, 169 unvaccinated participants were included, of which 40 were unwilling to receive the vaccine. The independent variables were basic information (age, gender), PHQ-2 ≥ 3 (positive screening for depressive mood), and GAD-2 ≥ 3 (positive screening for anxiety symptoms). A logistic regression analysis showed that screening positive for anxiety symptoms significantly influenced the unwillingness to be vaccinated (odds ratio = 3.157, 95% CI: 1.228–8.118) (Table 3).

## 4. Discussion

This study was conducted to determine the association between the psychological state of Japanese university students and their willingness to receive the COVID-19 vaccine. The findings of this study suggest that being unvaccinated against COVID-19 is associated with low perceived efficacy and importance of the vaccine. In addition, the presence of anxiety tendencies among the unvaccinated participants was shown to be associated with the willingness to receive the vaccine. These results partially supported our hypothesis. This is the first study to reveal the psychological state of people who are unwilling to receive the vaccine even when the vaccine is widely available in Japan. We were able to realistically assess their first impressions of the COVID-19 vaccine.

Most of the previous studies were conducted at the early stage of the pandemic before the vaccine became widely available to the general population. In a questionnaire survey on vaccination willingness conducted in September 2020, 66% of the respondents expressed their willingness to receive the vaccine [26]. Subsequently, the Japanese government began to promote the vaccine in February 2021 [27]; however, as many as 78% of people had already been vaccinated at least once in Japan by 11 October 2021 [11]. The results support previous studies that reported that willingness to be vaccinated changes over time [16,17]. Thus, to accurately understand the willingness of the population to be vaccinated, it was deemed necessary to conduct surveys both before and after the availability of vaccination services. Additionally, the vaccination coverage of young people (under 40) in Japan was reported to be 44.3% (14 October 2021) [11], whereas in this study it was 69.8%. This may be because the mean age of the participants was 21.4 years, and the age group was not exactly the same as that of previous reports, resulting in a high vaccination rate. Compared to previous studies of university students in other countries, the percentage of positive attitudes toward vaccination was similar or lower [15,28]. This may be related to the higher lack of trust in vaccines due to Japan’s original history [5].

In Model 1 (comparison by vaccination status), differences between the two groups were found in the perceived effectiveness of an existing vaccine and in its importance for protecting oneself and others. A study of vaccination intentions in African countries reported that knowledge about the importance of vaccination was associated with acceptance of the vaccination [21]. Thus, actively obtaining correct information from the government and the mass media can increase the knowledge and perceived importance of vaccines, leading to vaccine acceptance. Additionally, the present study showed that insufficient knowledge and underestimation of the importance of the vaccine were partially related to unvaccinated status.

There was no significant difference in psychological status between the vaccinated and unvaccinated groups (Model 1). Contrary to the findings of the current study, a previous study found that psychological factors affect vaccination acceptance [15,29]. One reason for this discrepancy may be the effect of the timing of the survey. Previous studies have shown that the intention to be vaccinated changes over time [16,17]. Besides, in Model 1, the unvaccinated group included the vaccination-willing participants, that is, those who intended to receive the vaccine in the future. One possible reason for this is that this survey was conducted at the beginning of the nationwide spread of the vaccination program, so there were still some issues to be resolved, such as the number of vaccines and environmental maintenance (securing vaccination sites and staff). In this study, 129 (76.3%) participants in the unvaccinated group were willing to receive the vaccine. In fact, the unvaccinated group was further divided into two subgroups, namely “Willing to receive the vaccine” and “Unwilling to receive the vaccine” (Model 2), and their psychological states were compared; those who were unwilling to receive the vaccine had higher indices of anxiety mood than those who were willing to receive the vaccine.

Furthermore, a logistic regression analysis revealed that the more anxious the participants were, the more unwilling they were to be vaccinated. This finding was similar to the results of a survey on vaccination intention conducted before the widespread availability of the vaccines [21,30], suggesting that the relationship between mental health and vaccination may remain the same despite the availability of vaccination services. However, the discussion on variables—prior to the availability of vaccination services—such as the perceived safety and efficacy of the vaccine, and the speculations about its side effects, may influence the willingness to be vaccinated [31,32,33]. These findings suggest that to increase vaccination intention and reduce the experience of anxiety among students, it is necessary to both educate them about the importance of vaccines and also create a mental health support system.

The first limitation of the current study is that the participants were given only two options to indicate their willingness to receive the COVID-19 vaccination (“willing” and “unwilling”). By increasing the number of options, it might be possible to obtain a more detailed understanding of the characteristics of those who were not willing to receive the vaccine. Second, because this was a cross-sectional study with an observational design, the relationship between the willingness to be vaccinated and actual future vaccination is unknown. Third, there may have been certain biases because the respondents were Japanese university students (selection bias), and anxiety and depression were assessed using screening instruments such as the GAD-2 and PHQ-2 (measurement bias). Fourth, the sample size of this study was very small compared to other similar large-scale surveys. The number of those who were unwilling to be vaccinated was lower than expected before designing the survey, and the ratio that we had originally expected (willing: unwilling to be vaccinated), about 1:1, turned out to be just above 3:1 (129:40). The reason for this is that the previous study was conducted before the start of the vaccination service (January 2021), and group vaccination had already started by the time of the present survey, so the number of subjects who were willing to receive the vaccination may have increased. It cannot be denied that the limitation of the sample size may have partially affected the results of this study. However, given the absence of any survey on the vaccination willingness of Japanese university students since vaccination services have become available, we believe that the data are valuable.

## 5. Conclusions

We surveyed Japanese university students using an online questionnaire to explore the association between their psychological status and their willingness to receive the COVID-19 vaccine. Our findings indicated that those with high anxiety were unwilling to receive the vaccine. This finding may be useful for informing vaccine dissemination, which is a key issue in the COVID-19 scenario, and new policy formations for infectious diseases. Moreover, the finding suggested the need for early education and mental health support for the students who did not receive the vaccine mainly because of anxiety symptoms. These results can guide the development of medical countermeasures against the pandemic and can be supplemented by future research including a variety of young people—not only students but also employed people.

## Figures and Tables

**Table 1:** Sociodemographic data, mental health status, and knowledge of and attitude toward COVID-19 among participants according to vaccination status.

Variable	Total (*n* = 560)	Vaccinated Group (*n* = 391)	Unvaccinated Group (*n* = 169)	*p*-Value	Effect Size
Age	21.4 ± 3.1	21.6 ± 3.4	21.0 ± 2.32	**0.029** ^a^	0.090
Gender				0.427 ^b^	0.034
Male	218 (38.9%)	148 (37.9%)	70 (41.4%)		
Female	342 (61.1%)	243 (62.1%)	99 (58.6%)		
Household structure				0.621 ^b^	0.041
Living alone	264 (47.1%)	185 (47.3%)	79 (46.8%)		
With others	296 (52.9%)	206 (52.7%)	90 (53.2%)		
Part-time job				0.753 ^b^	0.013
Employed	406 (72.5%)	285 (72.9%)	121 (71.6%)		
None	154 (27.5%)	106 (27.1%)	48 (28.4%)		
Movement restrictions				0.504 ^b^	0.049
Strict	56 (10.0%)	39 (10.0%)	17 (10.1%)		
Except for shopping or work	444 (79.3%)	314 (80.3%)	130 (76.9%)		
No	60 (10.7%)	38 (9.7%)	22 (13.0%)		
Fear of COVID-19 infection (Yes)	464 (82.9%)	325 (83.1%)	139 (82.3%)	0.802 ^b^	0.011
Knowledge about the COVID-19 vaccine (Yes)					
Can one be reinfected after recovering from COVID-19 infection?	535 (95.5%)	375 (95.9%)	160 (94.7%)	0.517 ^b^	0.027
Is there currently an effective vaccine against COVID-19?	413 (73.8%)	300 (76.7%)	113 (66.9%)	**0.015** ^b^	0.103
Importance of vaccination (Likert scale: 1–5)					
To protect self	5.0 [4.0–5.0]	5.0 [4.0–5.0]	4.0 [3.0–5.0]	**<0.001** ^a^	0.326
To protect others	5.0 [4.0–5.0]	5.0 [4.0–5.0]	4.0 [4.0–5.0]	**<0.001** ^a^	0.224
Anxiety symptoms (GAD-2 score ≥ 3)	85 (15.2%)	56 (14.3%)	29 (17.2%)	0.390 ^b^	0.036
Depressive symptoms (PHQ-2 score ≥ 3)	72 (12.9%)	54 (13.8%)	18 (10.7%)	0.305 ^b^	0.043

Note. Statistically significant results are in **bold**. GAD-2, generalized anxiety disorder. PHQ-2, Patient Health Questionnaire. GAD-2 and PHQ-2 scores of ≥3 are considered a positive screening. Data are expressed as means ± SD, *n* (%), or medians (interquartile range). ^a^ Results of the Mann–Whitney U test. ^b^ Results of the chi-squared test.

**Table 2:** Sociodemographic data, mental health status, and knowledge of and attitude toward COVID-19 among unvaccinated participants according to vaccination willingness.

	Unvaccinated Respondents (*n* = 169)			
Variable	Group A (*n* = 129)	Group B (*n* = 40)	*p*-Value	Effect Size
Age	21.0 ± 0.20	21.1 ± 0.37	0.528 ^a^	0.049
Gender			0.874 ^b^	0.012
Male	53 (41.1%)	17 (42.5%)		
Female	76 (58.9%)	23 (57.5%)		
Household structure			0.507 ^b^	0.090
Living alone	59 (45.7%)	20 (50.0%)		
With others	70 (54.3%)	20 (50.0%)		
Part-time job			0.290 ^b^	0.081
Employed	95 (73.6%)	26 (65.0%)		
None	34 (26.4%)	14 (35.0%)		
Movement restrictions			0.485 ^b^	0.092
Strict	12 (9.3%)	5 (12.5%)		
Except for shopping or work	102 (79.1 %)	28 (70.0%)		
No	15 (11.6 %)	7 (17.5%)		
Fear of COVID-19 infection (Yes)	109 (84.5 %)	30(75.0%)	0.170 ^b^	0.106
Knowledge about the COVID-19 vaccine (Yes)				
Can one be reinfected after recovering from COVID-19 infection?	124 (96.1%)	36 (90.0%)	0.132 ^b^	0.116
Is there currently an effective vaccine against COVID-19?	95 (73.6%)	18 (45.0%)	**0.001** ^b^	0.259
Importance of vaccination (Likert scale: 1–5)				
To protect self	4.0 [4.0–5.0]	3.0 [2.0–3.0]	**<0.001** ^a^	0.528
To protect others	5.0 [4.0–5.0]	3.0 [3.0–4.0]	**<0.001** ^a^	0.536
Anxiety symptoms (GAD-2 score ≥ 3)	17 (13.2%)	12 (30.2%)	**0.014** ^b^	0.033
Depression symptoms (PHQ-2 score ≥ 3)	13 (10.1%)	5 (12.5%)	0.664 ^b^	0.190

Note: Group A refers to the group with the participants who were willing to receive the vaccine. Group B refers to the group with the participants who were unwilling to receive the vaccine. Statistically significant results are in **bold**. GAD-2, generalized anxiety disorder. PHQ-2, Patient Health Questionnaire. Data are expressed as means ± SD, *n* (%), or medians (interquartile range). ^a^ Results of the Mann–Whitney U test. ^b^ Results of the chi-squared test.

**Table 3:** Logistic regression analysis for Model 2: association of the willingness to receive the vaccine with psychological distress assessment domains.

Primary Outcome	Unadjusted ^a^ OR (95% CI)	*p*-Value	Adjusted ^b^ OR (95% CI)	*p*-Value
Anxiety symptoms (GAD-2 score ≥ 3)	2.824 (1.210–6.587)	**0.016**	3.157 (1.228–8.118)	**0.017**

Statistically significant results are in **bold**. ^a^ Only anxiety symptoms (GAD-2 score ≥ 3) were considered for vaccination willingness. ^b^ Adjusted model adjusting for age, gender, and depressive symptoms (PHQ-2 score ≥ 3). GAD-2, generalized anxiety disorder. CI, confidence interval. Variance inflation factor: PHQ-2: 1.54; GAD-2: 1.54.

## Data Availability

The data presented in this study are available on request from the corresponding author. The data are not publicly available due to restrictions of privacy.
